# Targeting the FAK-Src Complex in Desmoplastic Small Round Cell Tumors, Ewing Sarcoma, and Rhabdomyosarcoma

**DOI:** 10.1155/2022/3089424

**Published:** 2022-05-11

**Authors:** Anke E. M. van Erp, Melissa H. S. Hillebrandt-Roeffen, Niek F. H. N. van Bree, Tim A. Plüm, Uta. E. Flucke, Ingrid M. E. Desar, Emmy D. G. Fleuren, Winette T. A. van der Graaf, Yvonne M. H. Versleijen-Jonkers

**Affiliations:** ^1^Department of Medical Oncology, Radboud University Medical Center, P.O. Box 9101, 6500 HB, Nijmegen, Netherlands; ^2^Department of Pathology, Radboud University Medical Center, P.O. Box 9101, 6500 HB, Nijmegen, Netherlands; ^3^Children's Cancer Institute Australia, Lowy Cancer Research Centre, University of NSW, Sydney, NSW, Australia; ^4^Department of Medical Oncology, Netherlands Cancer Institute-Van Leeuwenhoek, 1066 CX, Amsterdam, Netherlands

## Abstract

Desmoplastic small round cell tumors (DSRCTs), Ewing sarcoma (ES), and alveolar and embryonal rhabdomyosarcoma (ARMS and ERMS) are malignant sarcomas typically occurring at young age, with a poor prognosis in the metastatic setting. New treatment options are necessary. Src family kinase inhibitor dasatinib single-agent treatment has been investigated in a phase 2 study in patients with advanced sarcomas including ES and RMS but failed as a single agent in these subtypes. Since previous studies demonstrated high FAK and Src activities in RMS and ES tissue and cell lines, and dasatinib treatment was shown to upregulate activated FAK, we hypothesized that FAK-Src combination treatment could potentially be an interesting treatment option for these tumor types. We examined the effects of targeting the FAK-Src complex by addressing (p)FAK and (p)Src expressions in tumor sections of DSRCT (*n* = 13), ES (*n* = 68), ARMS (*n* = 21), and ERMS (*n* = 39) and by determining the antitumor effects of single and combined treatment with FAK inhibitor defactinib and multikinase (Abl/SFK) inhibitor dasatinib *in vitro* on cell lines of each subtype. *In vivo* effects were assessed in DSRCT and ERMS models. Concurrent pFAK and pSrc expressions (H-score >50) were observed in DSRCT (67%), ES (6%), ARMS (35%), and ERMS (19%) samples. Defactinib treatment decreased pFAK expression and reduced cell viability in all subtypes. Dasatinib treatment decreased pSrc expression and cell viability in each subtype. Combination treatment led to a complete reduction in pFAK and pSrc in each cell line and showed enhanced cell viability reduction, drug synergy, DNA damage induction, and a trend toward higher apoptosis induction in DSRCT, ERMS, and ARMS but not in ES cells. These promising *in vitro* results unfortunately do not translate into promising *in vivo* results as we did not observe a significant effect on tumor volume *in vivo*, and the combination did not show superior effects compared to dasatinib single-agent treatment.

## 1. Introduction

Sarcomas are rare tumors of the connective tissue with over 70 subtypes. Desmoplastic small round cell tumors (DSRCTs) are very rare (incidence of 0.2–0.5/million persons per year) and highly malignant soft tissue sarcomas (STS) mainly seen in adolescent and young adult (AYA) men [1, 2]. Ewing sarcoma (ES) is the second most common primary bone tumor in children [3]. Rhabdomyosarcoma (RMS) is the most common STS in pediatric patients but may also occur in adolescent and young adult patients. Alveolar and embryonal rhabdomyosarcoma (ARMS and ERMS) are the most frequent subtypes in children. Current treatment for each of these sarcoma subtypes consists of combination chemotherapy, when possible surgery, and on indication radiation therapy. Due to the similarities between DSRCTs and ES, DSRCT treatment is based on the ES chemotherapy regimen. DSRCT patients often present with widely disseminated disease at diagnosis, and despite intensive treatment, current 5-year overall survival (OS) rates remain approximately 15% [4]. The 5-year OS rates for localized ES and metastatic ES are 75% and 25%, respectively [3]. Survival rates for RMS are 88% (low-risk), 76% (intermediate-risk), and 25–40% (high risk; including fusion-positive RMS) [5, 6]. In addition, advanced age at the time of diagnosis correlates with decreased survival rates [7]. Despite intensive treatment with conventional chemotherapeutic agents, survival rates for patients with metastatic disease remain low for each of these subtypes (5-year OS:∼10–25%). Moreover, all survival rates have stagnated over the last decades and novel treatment options are, therefore, necessary.

Focal adhesion kinase (FAK) is a nonreceptor tyrosine kinase (non-RTK) involved in a myriad of cellular processes. FAK acts as both a signaling and a scaffolding protein. The signaling capacities of FAK are dependent on the phosphorylation of several kinase domains (Tyr397, Tyr567, and Tyr577) and often involve interaction with Src, a non-RTK known to be of importance in sarcoma [8]. Upon activation, FAK is phosphorylated at the autophosphorylation site Tyr397. In addition to kinase activity, Tyr397 phosphorylation acts as a high-affinity binding site for Src family kinases (SFK), such as Src. Src activity is mediated by its autophosphorylation domain Tyr416 [9]. Upon binding, Src activates the Tyr567 and Tyr577 domains of FAK, resulting in a fully activated FAK. The FAK-Src complex can subsequently activate further downstream signaling pathways, resulting in the activation of processes involved in cellular proliferation, survival, invasion, migration, and cancer stem cell activity [10]. FAK and Src overexpressions have been observed in a variety of tumor types and correlated with invasive and metastatic disease, and patient outcome (reviewed in [10, 11]). In addition, previous phosphoproteomic screening of ES and RMS cell lines and tumor tissue showed high phosphorylation of FAK [12, 13]. Moreover, ES and RMS cell lines showed concurrent phosphorylation of FAK and Src, making the FAK-Src complex a potential target for treatment in these sarcoma subtypes [12, 14]. In accordance with the observed pFAK and pSrc expression levels, *in vitro* and *in vivo* antitumor effects following single-agent FAK and Src inhibition were reported in ES and RMS [12, 13, 15–18]. Dasatinib single-agent treatment has been investigated in a phase 2 study (SARC009) in patients with advanced sarcomas including ES and RMS but failed as a single agent in these subtypes [19]. Single-agent defactinib could also have limited clinical effects in DSRCT, ES, and RMS patients as seen previously in non-small cell lung cancer [20]. In hepatocellular carcinoma, it was shown that dasatinib treatment induced upregulation of activated FAK [21]. Therefore, we specifically examined the effects of targeting the FAK-Src complex by addressing pFAK and pSrc expressions in clinical tumor sections of primary and post-treatment resections, metastatic and locally recurrent DSRCT (*n* = 13), ES (*n* = 68), and RMS (ARMS *n* = 21, ERMS *n* = 39) and by determining the antitumor effects of the combined treatment of the FAK inhibitor defactinib and the multikinase (Abl/SFK) inhibitor dasatinib in DSRCT, ES, ARMS, and ERMS cell lines. Effects of defactinib and dasatinib combination treatment were assessed in an *in vivo* DSRCT and ERMS model.

## 2. Materials and Methods

### 2.1. Immunohistochemistry (IHC)

Tissue microarrays (TMAs) containing primary and post-treatment resections, metastatic or locally recurrent tumor tissue of DSRCT (*n* = 13), ES (*n* = 68), ARMS (*n* = 21), and ERMS (*n* = 39) were stained for baseline FAK, Src, phosphorylated FAK Tyr397 (pFAK), and phosphorylated Src Tyr416 (pSrc) expression. pFAK expression could be evaluated in 13/13 DSRCT, 68/68 ES, 20/21 ARMS, and 39/39 ERMS tissues sections. pSrc expression could be evaluated in 12/13 DSRCT, 64/68 ES, 21/21 ARMS, and 37/39 ERMS tissue sections. Patient characteristics are presented in Table 1. A detailed description of the methodology and statistical analysis can be found in the Supplementary Material.

### 2.2. Cell Culture and Compounds

The JN-DSRCT-1 cell line (EWSR1-WT1) was generously provided by Dr. Janet Shipley (Institute of Cancer Research, UK). The EW8 (Ewing sarcoma EWSR1-FLI1), RD (ERMS), Rh18 (ERMS), Rh30 (ARMS PAX3-FOXO1A), and Rh41 (ARMS, PAX3-FOXO1A) cell lines were generously provided by Dr. Peter Houghton (Pediatric Preclinical Testing Program, USA), and the TC32 cell line (Ewing sarcoma, EWSR1-FLI1) was generously provided by Dr. Friederike Meyer-Wentrup (Princess Maxima Center for Pediatric Oncology, Utrecht, Netherlands).

The JN-DSRCT-1 was cultured in DMEM : F12 GlutaMAX™ medium (Gibco, Thermo Fisher, Breda, NL). EW8, Rh41, and Rh30 were cultured in RPMI 1640 medium (Lonza, Westburg, Leusden, NL). RD and TC32 were cultured in DMEM (Lonza) and Rh18 in McCoy's 5 A medium (Lonza). All culture media were supplemented with 10% fetal bovine serum (Gibco) and 0.1% gentamycin or 1% penicillin/streptomycin (Lonza). Cells were cultured in a humidified atmosphere of 5%CO_2_/95% air at 37°C.

The FAK inhibitor defactinib and Abl/SFK inhibitor dasatinib were purchased from Selleckchem (Houston, TX, USA) and diluted in DMSO for *in vitro* experiments. Defactinib and dasatinib were diluted in 0.5% hydroxypropyl methylcellulose (HPMC)/0.2% Tween-80 in sterile water and 9.5% DMSO/5.1% PEG-300/5.1% Tween-80 in sterile water [22] for *in vivo* experiments, respectively.

### 2.3. Cell Viability Assay

Cell viability was assessed by the MTS (3-(4,5-dimethylthiazol-2-yl)-5-(3-carboxymethoxyphenyl)-2-(4-sulfophenyl)-2H-tetrazolium) assay. Cells were treated with increasing drug concentrations for 72 h (TC32, EW8, Rh30, and RD), 120 h (JN-DSRCT-1 and Rh41), or 144 h (Rh18), based on the estimated cell division rate. MTS solution (CellTiter 96 Aqueous Solution Cell Proliferation Assay, Promega, WI, USA) was added (10 *μ*l), and plates were incubated for 2 h at 37°C. Extinction was measured at 490 nm (iMark microplate absorbance reader, Bio-Rad, CA, USA). IC_50_ values were calculated with GraphPad Prism version 5.03 software.

### 2.4. Combination Treatment

Cell viability following simultaneous combination treatment of defactinib and dasatinib was assessed, and drug synergy was calculated as previously described [23]. All drug concentrations were combined in a constant ratio expressed in a fraction or a multiplication of the IC_50_ concentration, and the combination index (CI) and dose reduction index (DRI) were calculated using CompuSyn software [24]. CI < 1.0, CI = 1.0, and CI > 1.0 represent synergistic, additive, and antagonistic effects, respectively. DRI >1.0 indicates a favorable dose reduction of the drug in the combination treatment. Drug synergy is represented in an isobologram. The X- and Y-axes represent the fraction of the portion of the drug in the combination treatment (D_1_ + D_2_) necessary to reduce x% cell viability (D_X_)_1/2_ divided by the dose necessary as a single agent to reduce the same x% cell viability (D_1/2_). D1 = defactinib, and D2 = dasatinib.

### 2.5. Western Blot

Western blot analysis was performed as previously described [23]. Cells were treated for 24 h with IC_50_-based single-agent or combination treatment. A detailed description of each antibody can be found in the Supplementary Material.

### 2.6. Apoptosis Assay

The level of apoptotic cells following 24–48 h IC_50_-based single-agent and combination treatment was measured using the annexin V/propidium iodide (PI) double-staining apoptosis assay (BioVision, CA, USA). Detached and adhering cells were collected and subsequently incubated with annexin V-FITC and PI in a CaCl_2_-enriched culture medium. The number of apoptotic cells (annexin V^+^) was measured on the CytoFLEX flow cytometer (Beckman Coulter, CA, USA) and calculated using FlowJo version 10.0.

### 2.7. In Vivo Combination Treatment

All applicable international, national, and institutional guidelines for the care and use of animals have been followed. All procedures performed were in accordance with the ethical standards of the animal ethical committee of Radboud University, Nijmegen, Netherlands (project^#^ 2015-0109).

A total of 5 × 10^6^ JN-DSRCT-1 or RD cells were subcutaneously injected in a 1 : 1 culture medium: Matrigel® matrix (Corning, NY, USA) solution, in male (JN-DSRCT-1) and female (RD) SCID mice (6–8 weeks of age). The experiment was started at a tumor size of approximately 0.25–0.4 cm^3^, and the animals were randomly allocated to the treatment groups (5 mice per group). Defactinib and dasatinib were administered at 50 mg/kg/day for 21 (RD) or 28 (JN-DSRCT-1) days. Tumor growth was monitored by caliper measurements twice weekly and depicted as relative tumor volume (RTV) as previously described [23]. Tumor viability (%) (H & E staining) was assessed by an expert pathologist (UF) and caspase-3 (apoptosis), *ƴ*H2AX, (p)FAK, and (p)Src expressions were assessed by IHC in the remaining viable tumor tissue as previously described and in Supplementary Materials [23].

## 3. Results

### 3.1. pFAK and pSrc Expressions in Clinically Derived Tumor Tissue

Baseline FAK, Src, pFAK (Tyr397), and pSrc (Tyr416) expressions were assessed in the tumor tissue of primary and post-treatment resections, metastatic or locally recurrent DSRCT (*n* = 13), ES (*n* = 68), ARMS (*n* = 21), and ERMS (*n* = 39). The number of samples can vary between the stainings since not all samples were evaluable for each staining. An example per staining intensity is depicted in Supplementary Figure S1.

Baseline FAK expression (H-score >0) was seen in 92% of DSRCT (12/13), 85% of ES (52/61), 70% of ARMS (14/20), and 61% (22/36) of ERMS tissues.

**Figure 1:**
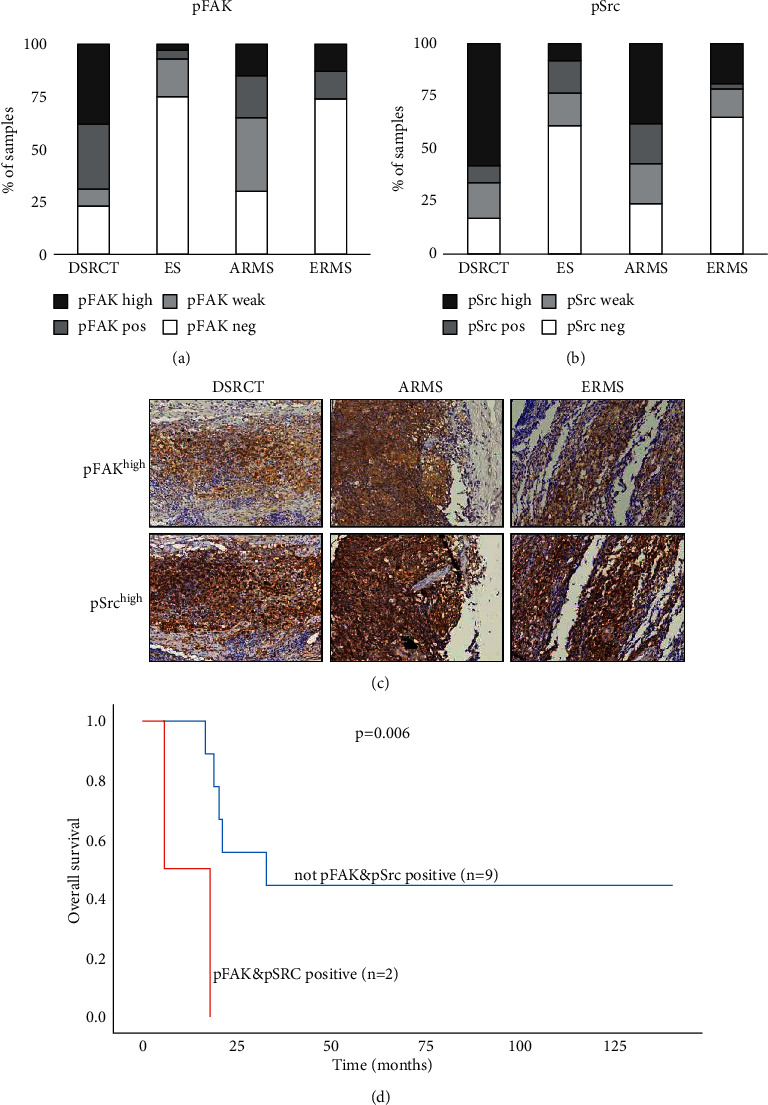
pFAK (Tyr397) and pSrc (Tyr416) expressions in DSRCT, ES, ARMS, and ERMS tumor tissue. (a) pFAK expression in primary and post-treatment resections, and metastatic or locally recurrent DSRCT (*n* = 13), ES (*n* = 68), ARMS (*n* = 20), and ERMS (*n* = 39) tumor tissue assessed by immunohistochemistry. (b) pSrc expression in primary and post-treatment resections, and metastatic or locally recurrent DSRCT (*n* = 12), ES (*n* = 64), ARMS (*n* = 21), and ERMS (*n* = 37). pFAK and pSrc expressions are subdivided into negative (H-score 0; pFAK/pSrc^neg^), weak-positive (H-score ≤50; pFAK/pSrc^pos^), positive (H-score 51–100; pFAK/pSrc^pos^), and high-positive expression (H-score >100; pFAK/pSrc^high^). (c) Example of a pFAK^high^ and pSrc^high^ double-positive DSRCT, ARMS, and ERMS tumor tissue. Images taken at x 80 magnification. (d) Kaplan-Meier survival analysis of overall survival according to concurrent positive pFAK and pSrc expression in ARMS.

The pFAK expression (H-score >0) was seen in 77% of DSRCT (10/13), 25% of ES (17/68), 70% of ARMS (14/20), and 26% of ERMS tissues (10/39). High pFAK expression (H-score >100) was seen in 38% of DSRCT (5/13), 3% of ES (2/68), 15% of ARMS (3/20), and 13% of ERMS (5/39) (Figure 1(a)).

Baseline Src expression was seen in 100% of DSRCT (13/13), 88% of ES (56/64), 95% of ARMS (19/20), and 86% of ERMS (32/37). pSrc expression (H-score >0) was seen in 83% of DSRCT (10/12), 39% of ES (25/64), 76% of ARMS (16/21), and 35% of ERMS tissues (13/37). High pSrc expression (H-score >100) was seen in 58% of DRSCT (7/12), 8% of ES (5/64), 38% of ARMS (8/21), and 19% of ERMS (7/37) (Figure 1(b)).

Concurrent pFAK and pSrc expressions could be assessed in 12 DSRCT, 62 ES, 20 ARMS, and 36 ERMS tumor samples. Concurrent pFAK^pos^ and pSrc^pos^ expressions were seen in 8/12 (67%) DSRCT, 4/62 (6%) ES, 7/20 (35%) ARMS, and 7/36 (19%) ERMS tumor tissues (Table 2). Of these samples, 4/8 DSRCT, 0/4 ES, 3/7 ARMS, and 4/7 ERMS tumor tissues showed concurrent pFAK^high^ and pSrc^high^ expressions (Table 2, Figure 1(c)). pFAK and pSrc expressions showed a significant correlation in DSRCT (H-score >50, *p*=0.045), ES (H-score >50, *p*=0.002), ARMS (H-score >100, *p*=0.049), and ERMS tumor tissue (H-score >50, *p* ≤ 0.001; H-score >100, *p*=0.003) (Table 2). Positive pFAK and concurrent positive pFAK and pSrc expressions (both *p*=0.006) were significantly associated with a lower overall survival in ARMS patients (Figure 1(d)). pFAK, pSrc, or concurrent pFAK and pSrc expressions did not correlate with patient characteristics or event-free survival (EFS) and did not differ between primary, post-treatment, metastatic, or recurrent DSRCT, ARMS, ERMS, and ES tumor samples (data not shown).

### 3.2. Single-Agent Defactinib and Dasatinib Treatment

**Figure 2:**
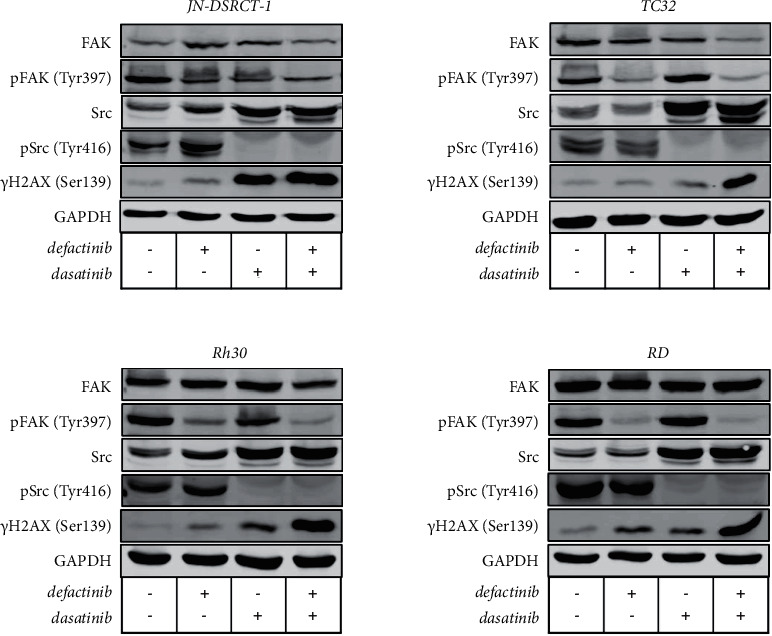
(p)FAK, (p)Src expression, and DNA damage (*γ*H2AX) following defactinib and dasatinib combination treatment in DSRCT, ES, ARMS, and ERMS cell lines. FAK, pFAK (Tyr397), Src, pSrc (Tyr416), *γ*H2AX (Ser139), and GAPDH expressions at baseline, post-24-h single-agent defactinib or dasatinib treatment, and post-24-h combination treatment with defactinib and dasatinib in the JN-DSRCT-1, TC32, Rh30, and RD cell lines assessed by Western blot analysis.

**Figure 3:**
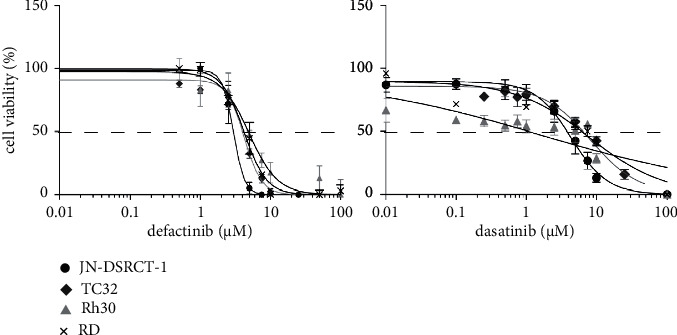
Cell viability following single-agent defactinib and dasatinib treatment in DSRCT, ES, ARMS, and ERMS cell lines. Cell viability (%) following single-agent defactinib and dasatinib treatment in the JN-DSRCT-1, TC32, Rh30, and RD cell lines. The dotted line represents the IC50 value.

The baseline expression levels of pFAK (Tyr397) and pSrc (Tyr416) in DSRCT, ES, ARMS, and ERMS cell lines and the effects of defactinib and dasatinib treatment on cell viability were determined. Two cell lines per subtype were used, except for DSRCT, for which only one established cell line was available. All cell lines showed pFAK and pSrc expressions, with low pSrc expression in the Rh18 cell line (Figure 2/S2A). Defactinib and dasatinib treatment inhibited cell viability with IC_50_ values ranging from 2.5 to 6.4 *μ*M and 0.6 to 7.7 *μ*M, respectively (Table 3). Cell viability was reduced in a dose-dependent manner in all cell lines, except for dasatinib treatment in the Rh30 cell line (Figure 3/S2B). Dasatinib treatment led to a sloping decrease in cell viability, with approximately 50% cell viability reduction starting at 0.6 *μ*M treatment.

### 3.3. Defactinib and Dasatinib Combination Treatment

#### 3.3.1. pFAK and pSrc Expressions

To investigate whether targeting the FAK-Src complex could increase the observed therapeutic responses after single-agent treatment, we investigated the effects of simultaneous defactinib and dasatinib combination treatment on (p)FAK and (p)Src expressions in one representative cell line per sarcoma subtype: JN-DSRCT-1, TC32, Rh30, and RD. Western blot results are shown in Figure 2 with quantification in Figure S3 (a) (d)). Dasatinib single-agent treatment and combination treatment led to a complete reduction of pSrc in each cell line. Defactinib single-agent treatment and combination treatment led to a clear reduction of pFAK in the Rh30, RD, and TC32 cell lines. JN-DSRCT-1 showed a more modest reduction in pFAK after single-agent defactinib treatment and combination treatment. Total FAK expression did not notably change upon treatment. Total SRC expression appeared to increase after dasatinib and combination treatment.

#### 3.3.2. Cell Viability and Drug Synergy

**Figure 4:**
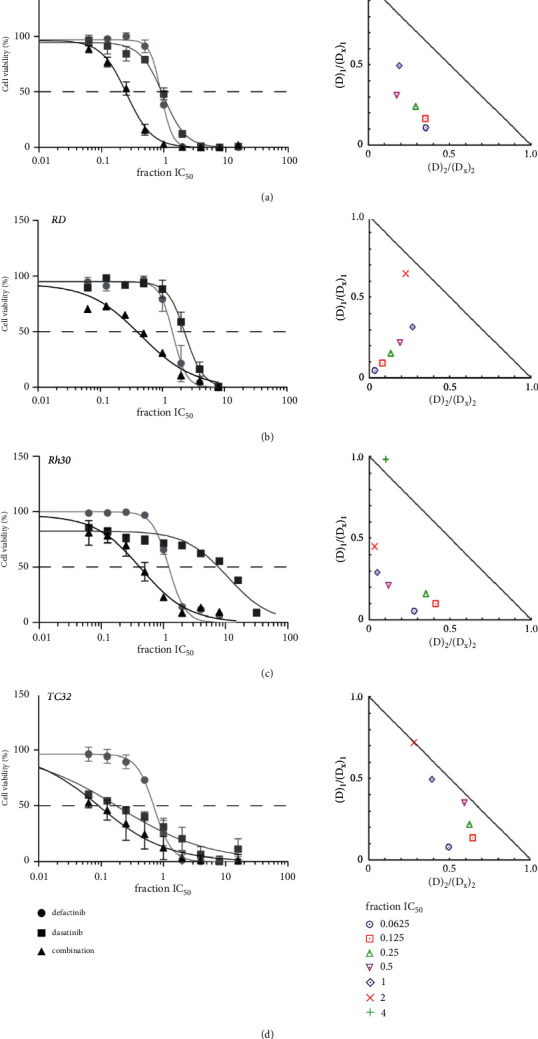
Cell viability and drug synergy following defactinib and dasatinib combination treatment in DSRCT, ERMS, ARMS, and ES cell lines. (a–d) Cell viability (%) following defactinib and dasatinib simultaneous, constant-ratio combination treatment in the JN-DSRCT-1 (a), RD (b), Rh30 (c), and TC32 (d) cell lines alongside the corresponding isobologram, representing the level of drug synergy. The X- and *Y*-axes of the isobologram represent the fraction of the portion of the drug in the combination treatment (D1 + D2) necessary to reduce an x% cell viability (D1/2) divided by the dose necessary as a single agent to generate a reduction of a similar x% cell viability (DX)1/2. D1 = defactinib, D2 = dasatinib. The line connecting the X- and *Y*-axes represents an additive effect (CI = 1). Points left of the line are considered a synergistic effect (CI < 1.0).

Cell viability analysis showed a shift toward a higher antitumor effect for the combination treatment compared to the respective single-agent treatments in the DSRCT, ERMS, and ARMS cell lines (Figures 4(a)–4(c), S4(a-b)). The ES cell lines, TC32 and EW8, did not show an enhanced cell viability reduction following the combination treatment compared to the single-agent treatments (Figure 4(d)/S4(c)). Drug synergy (CI < 1.0) and a favorable DRI (DRI >1.0) were observed for the combination treatments using concentrations equal to or below 2 × IC_50_ value in the DSRCT, ERMS, and ARMS cell lines. ES cell lines showed a more additive effect following combination treatment (CI∼1.0) (Figure 4 (S4(a-c), Table 4/S1).

#### 3.3.3. DNA Damage

The level of DNA damage was examined following 24-h combination treatment in one cell line per subtype selected on the most pronounced shift in cell viability following combination treatment compared to the respective single-agent treatments (JN-DSRCT-1, RD, Rh30, and TC32). Phosphorylated H2AX (*γ*H2AX) expression, indicative of DNA damage, following combination treatment was increased compared to the single-agent treatments, especially in the TC-32, RD, and Rh30 cell lines (Figure 2/S3(e)).

#### 3.3.4. Apoptosis

**Figure 5:**
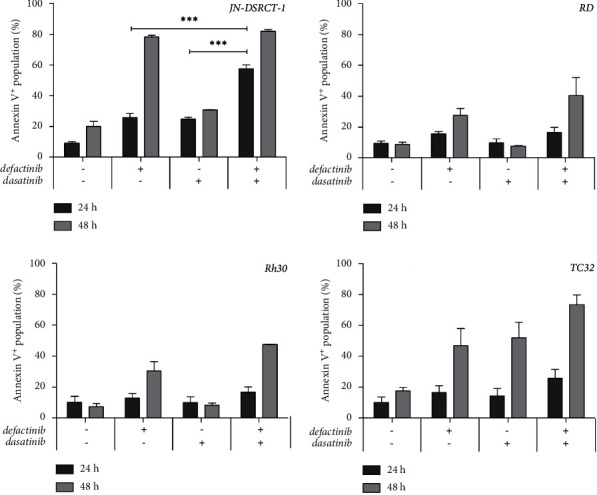
*Apoptosis* induction following defactinib and dasatinib combination treatment in DSRCT, ERMS, ARMS, and ES cell lines. The level of apoptotic cells (%; annexin V+) following 24 h and 48 h vehicle, single-agent defactinib, single-agent dasatinib, and defactinib and dasatinib combination treatment in the JN-DSRCT-1, RD, Rh30, and TC32 cell lines. ^∗∗∗^*p*-value <0.001.

Twenty-four-hour single-agent defactinib (26%) and dasatinib (25%) treatment significantly increased apoptosis compared to vehicle-treated cells (9%) in JN-DSRCT-1 cells (*p*-value <0.01; not shown). In addition, JN-DSRCT-1 cells showed a significant increase in apoptosis following 24-h combination treatment (60%) compared to defactinib (26%) and dasatinib (25%) single-agent treatment. No significant increase in apoptosis could be observed in the RD, Rh30, and TC32 cells following 24 h single-agent defactinib treatment (Figure 5; 24 h). Prolonged treatment (48 h) with defactinib showed an increase in apoptotic cells in all cell lines (Figure 5). Dasatinib treatment (48 h) also increased apoptosis in the TC32 cell line. Dasatinib treatment did not induce apoptosis in either of the RMS cell lines. Forty-eight-hour combination treatment induced apoptosis in each cell line, although not significantly different compared to defactinib, and in TC32 cells, dasatinib single-agent treatment (Figure 5).

### 3.4. In Vivo Defactinib and Dasatinib Combination Treatment in a DSRCT and ERMS Model

**Figure 6:**
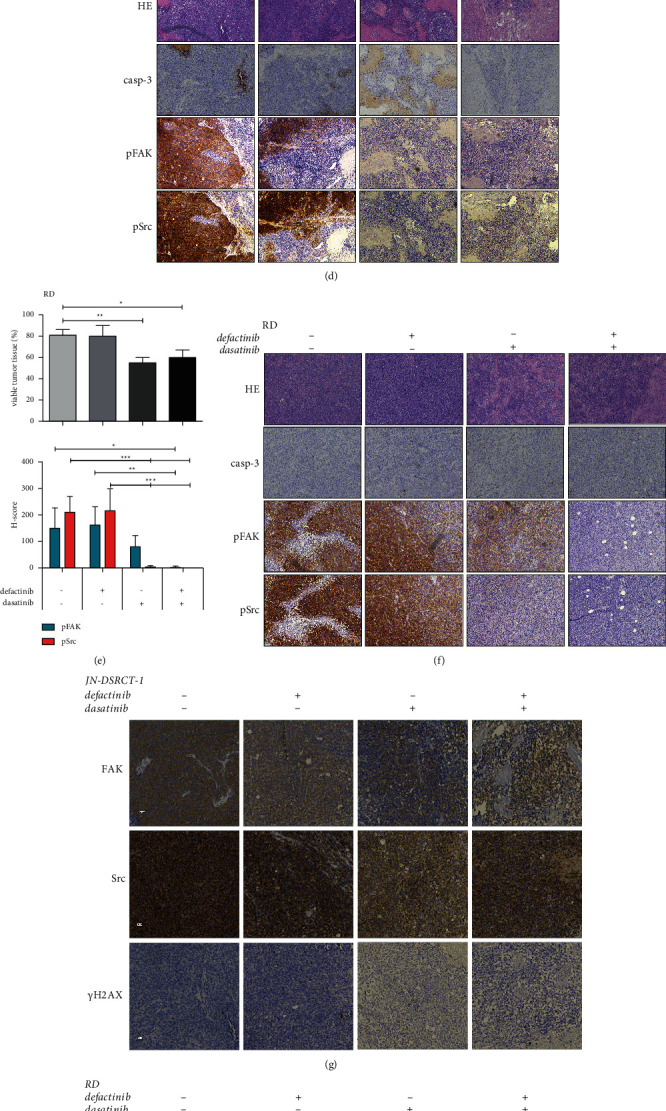
In vivo assessment of defactinib and dasatinib combination treatment in a DSRCT and ERMS model. (a) Relative tumor volume (RTV) following 28 days of vehicle, defactinib (50 mg/kg/day), dasatinib (50 mg/kg/day), and combination treatment in the JN-DSRCT-1 in vivo model. (b) Relative tumor volume (RTV) following 21 days of vehicle, defactinib (50 mg/kg/day), dasatinib (50 mg/kg/day), and combination treatment in the RD in vivo model. (c–f) The level of viable tumor tissue (HE), caspase-3 (casp-3), pFAK, and pSrc expressions following vehicle, single-agent defactinib, single-agent dasatinib, and combination treatment in vivo in the JN-DSRCT-1 (c-d) and RD (e-f) models. The differences between pFAK and pSrc expression showed similar significance levels (i.e., *p*-value <0.001) in the JN-DSRCT-1 model compared to the RD model. One representative line and asterisks are given. The level of FAK, Src, and ƴH2AX expressions following vehicle, single-agent defactinib, single-agent dasatinib, and combination treatment in vivo in the JN-DSRCT-1 (g) and RD (h) models. The differences between FAK, Src, and *ƴ*H2AX expressions between treatment groups in both models (i). ^*∗*^*p*-value <0.05, ^*∗∗*^*p*-value <0.01, and ^*∗∗∗*^*p*-value <0.001. HE: hematoxylin and eosin staining.

The DSRCT and ERMS cells showed the largest potential benefit from targeting the FAK-Src complex *in vitro* by showing a pronounced cell viability reduction following combination treatment (Figures 4(a) and 4(b)). Therefore, DSRCT and ERMS models were used to address the antitumor effects of defactinib and dasatinib combination *in vivo.* Neither single-agent treatment nor the combination treatment affected relative tumor volume (RTV) in the DSRCT and ERMS models (Figures 6(a) and 6(b)). The DSRCT model only showed a very modest growth impeding the visualization of effects on tumor volume in this model. Treatment duration was reduced to 21 days for the RD model due to an exponential growth of the tumor, resulting in skin ulcerations, an endpoint for the experiment. In contrast to the lack of changes in RTV, the amount of viable tumor tissue was decreased following dasatinib and combination treatment compared to the vehicle and defactinib-treated group in both subtypes, with significant differences for defactinib versus dasatinib treatment in the DSRCT model and vehicle versus dasatinib and combination treatment in the RD model. No difference in tissue viability was observed between the dasatinib- and the combination-treated group and caspase-3 expression (data not shown) in the remaining viable tumor tissue that did not differ between the different treatment groups (Figures 6(c)-6(f)). FAK and Src expressions did not change after treatment in the JN-DSRCT model (Figures 6(g)–6(i)). pFAK and pSrc expressions showed a (nearly) complete reduction following single-agent dasatinib and combination treatment in the DSRCT model (Figures 6(c)–6(d)). Both FAK and Src expressions were still present but lower after single-agent dasatinib treatment in the RD model (both *p*=0.03) (Figures 6(h)–6(i)). pFAK expression was still present following single-agent dasatinib treatment but completely reduced following combination treatment. In addition, a complete reduction in pSrc could be observed following single-agent dasatinib and combination treatment (Figures 6(e)–6(f)).

Phosphorylated H2AX (*γ*H2AX) expression was predominantly seen in the RD model and was significantly higher in the dasatinib-treated tumors compared to the vehicle group (*p*=0.03) (Figures 6(h)–6(i)).

## 4. Discussion

FAK and Src inhibition, either as a single agent or as part of a combination treatment, has previously been shown to have preclinical effects in a variety of tumor types [21, 25–37]. In this study, we specifically examined the effects of targeting the FAK-Src complex in pediatric and AYA sarcomas by examining pFAK and pSrc expressions in clinically derived tumor material of DSRCT, ES, ARMS, and ERMS patients and by examining *in vitro* and *in vivo* effects of the FAK inhibitor defactinib and the Abl/SFK inhibitor dasatinib combination treatment.

Concurrent positive pFAK and pSrc expressions were observed in 67% of DSRCT, 6% of ES, 35% of ARMS, and 19% of ERMS tumor tissue. This suggests that the FAK-Src complex might be a target for treatment in a subgroup of patients. Moreover, positive pFAK and concurrent positive pFAK and pSrc expressions are significantly associated with a lower overall survival in ARMS patients. Nevertheless, the analysis of a larger cohort is needed to verify the level and the prognostic value of concurrent pFAK and pSrc expressions in DSRCT, ES, ARMS, and ERMS.

In line with previous findings, single-agent treatment with defactinib or dasatinib led to a dose-dependent decrease in cell viability in the ERMS, ARMS, and ES cell lines [12, 13, 15, 16]. We now also showed, for the first time, positive results in the JN-DSRCT-1 cell line. Moreover, *in vitro* treatment with the combination of defactinib and dasatinib showed drug synergy in the DSRCT, ERMS, and ARMS cells. A similar synergistic effect was previously observed in neuroblastoma, acute lymphoblastic leukemia, and hepatocellular carcinoma (HCC) cells [21, 27, 32]. DSRCT, ERMS, and ARMS cells showed a higher level of drug synergy compared to ES cells. This could probably be explained by the observation that ES cells already showed a clear increase in apoptosis after single defactinib and single dasatinib treatment. We do realize that we may miss the variability between cell lines because of the choice to examine the combination effects in only one cell line per sarcoma subtype.

We showed a clear decrease in pFAK after defactinib treatment and in pSrc after dasatinib treatment and a clear reduction in both pFAK and pSrc after combination treatment as expected. Total Src expression appeared to increase after dasatinib and combination treatment, which was unexpected but also shown in previous studies [38–41].

*In vivo* analysis of simultaneous combination treatment did not show an effect on relative tumor volume. For the DSRCT model, this could be due to its very modest growth impeding the visualization of effects on tumor volume. The RD model on the other hand showed an exponential growth, which may have complicated treatment. We did show a reduction in tumor viability post dasatinib and combination treatment. Based on these findings, it can be suggested that, similar to pazopanib efficacy in advanced STS, additional measurements of tumor metabolism using radiological markers could potentially lead to a better representation of dasatinib and defactinib treatment efficacy [42]. Tumor viability reduction was similar in both single-agent dasatinib and combination treatment groups, and phosphorylation of H2AX was significantly higher in the tumors treated with dasatinib. It is known that dasatinib can induce DNA damage [43]. The *in vivo* results revealed that the combination treatment does not show superior effects compared to dasatinib single-agent treatment as shown *in vitro*. The superior *in vivo* effect of dasatinib could potentially be explained by the anti-angiogenic properties of dasatinib [44].

However, dasatinib single-agent treatment was already investigated in a phase 2 study in patients with advanced sarcomas and failed as a single agent in most sarcoma subtypes, including Ewing sarcoma and rhabdomyosarcoma [19]. Other combination strategies with dasatinib should, therefore, be considered. Since dasatinib has been shown to synergize with immune checkpoint inhibition in non-small cell lung cancer models, this could be a promising combination for future research [45].

The effects of FAK-Src targeting in this study may presumably be underestimated because of the two-dimensional culture of cells in the absence of the extracellular matrix.

In addition, the role of the FAK-Src complex in tumor cell invasion and cell-cell adhesion suggests that *in vivo* combination treatment focused on tumor migration and tumor outgrowth at the metastatic site could potentially be of interest for further research [10].

## 5. Conclusions

In conclusion, concurrent pFAK and pSrc expressions are present in a subset of DSRCT, ES, ARMS, and ERMS tumor tissue. This, in combination with the reduction in cell viability, induction of DNA damage and increased apoptosis following defactinib and dasatinib combination treatment in DSRCT, ERMS, and ARMS cells showed that targeting of the FAK-Src complex could enhance the antitumor effect in these sarcoma subtypes. These promising *in vitro* results unfortunately do not translate into promising *in vivo* results as we did not observe significant effects on tumor volume and did not find superior effects of the combination *in vivo* compared to dasatinib single-agent treatment. Therefore, these results do not yet encourage further clinical research into the therapeutic potential of this combination treatment in DSRCT, ARMS, and ERMS.

## Figures and Tables

**Table 1:** Patient characteristics of ES, DSRCT, ARMS, and ERMS tumor tissue.

*Tumor type*	*Characteristics*		*N (%)*
DSRCT (n = 13)	Gender	Male	9 (69)
		Female	4 (31)
	Age at diagnosis	<18 y ≥18 y	4 (31) 9 (69)
	Translocation	EWSR1-WT1	13 (100)
	Metastases	Yes	10 (77)
		Unknown	3 (23)
	Initial metastases	Yes	10 (100)^a^
	Sample source	Primary tumor	9 (69)
		Resection (post-treatment)	4 (31)

ES (*n* = 68)	Gender	Male	31 (46)
		Female	37 (54)
	Age at diagnosis	<18 y	44 (65)
		≥18 y	24 (35)
	Translocation	EWSR1-FLI1	56 (82)
		EWSR1-ERG	9 (13)
		Positive (not specified)	3 (5)
	Metastases	Yes	19 (28)
		No	30 (44)
		Unknown	19 (28)
	Initial metastases	Yes	7 (37)^a^
	Sample source	Primary tumor	33 (49)
		Resection (post-treatment)	18 (26)
		Metastasis	11 (16)
		Local recurrence	4 (6)
		Other	2 (3)

ARMS (*n* = 21)	Gender	Male	13 (62)
		Female	8 (38)
	Age at diagnosis	<18 y	15 (71)
		≥18 y	6 (29)
	Translocation	PAX3-FOXO1	10 (48)
		PAX7-FOXO1	5 (24)
		Positive (not specified)	6 (28)
	Metastases	Yes	9 (43)
		No	3 (14)
		Unknown	9 (43)
	Initial metastases	Yes	3 (33)^a^
	Sample source	Primary tumor	7 (33)
		Lymph node	7 (33)
		Metastasis	1 (5)
		Resection (post-treatment)	3 (14)
		Local recurrence	3 (14)

ERMS (*n* = 39)	Gender	Male	35 (90)
		Female	4 (10)
	Age at diagnosis	<18 y	35 (90)
		≥18y	4 (10)
	Metastases	Yes	2 (5)
		No	33 (85)
		Unknown	4 (10)
	Initial metastases	Yes	1 (50)^a^
	Sample source	Primary tumor	31 (80)
		Resection (post-treatment)	6 (15)
		Metastasis	2 (5)

DSRCT: desmoplastic small round cell tumor; ES: Ewing sarcoma; ARMS: alveolar rhabdomyosarcoma; ERMS: embryonal rhabdomyosarcoma; N: number of patients; a percentage calculation: (total with initial metastases/total with metastases) ^*∗*^100%.

**Table 2:** Concurrent positive and high-positive pFAK and pSrc expressions in DSRCT, ES, ARMS, and ERMS tumor tissue.

Tumor type	pFAK^pos^ and pSrc^pos^	pFAK^high^ and pSrc^high,a^	*p*-value (pFAK^pos^ and pSrc^pos^)	*p*-value (pFAK^high^ and pSrc^high^)
DSRCT (*n* = 12)	8/12	4/8	0.045	N.S.
ES (*n* = 62)	4/62	0/4	0.002	N.S.
ARMS (*n* = 20)	7/20	3/7	N.S.	0.049
ERMS (*n* = 36)	7/36	4/7	≤0.001	0.003

DSRCT: desmoplastic small round cell tumor; ES : Ewing sarcoma; ARMS: alveolar rhabdomyosarcoma; ERMS: embryonal rhabdomyosarcoma; FAK: focal adhesion kinase; a pFAKhigh and pSrchigh tumors presented as a fraction of the pFAKpos and pSrcpos group; p-value: correlation between pFAK and pSrc expression as calculated by Fisher's exact test; N.S.: not statistically significant.

**Table 3:** IC_50_ value defactinib and dasatinib in DSRCT, ES, and RMS cell lines.

Tumor type	Cell line	Defactinib (*μ*M) (mean ± SD)	Dasatinib (*μ*M) (mean ± SD)
DSRCT	JN-DSRCT-1	2.5 ± 1.1	5.4 ± 1.0
ES	TC32	3.7 ± 0.7	7.7 ± 1.7
	EW8	4.6 ± 1.9	6.3 ± 2.7
ARMS	Rh30	4.3 ± 0.3	0.6 ± 0.04
	Rh41	4.1 ± 0.7	6.4 ± 0.4
ERMS	RD	4.5 ± 1.3	6.9 ± 2.9
	Rh18	6.4 ± 1.3	4.8 ± 0.9

DSRCT: desmoplastic small round cell tumor; ES : Ewing sarcoma; ARMS: alveolar rhabdomyosarcoma; ERMS: embryonal rhabdomyosarcoma; SD: standard deviation

**Table 4:** FA, CI, and DRI values for defactinib and dasatinib combination treatment in DSRCT, ES, ARMS, and ERMS cell lines.

Subtype	Cell line	Fraction IC50	FA-value (mean ± SD)	CI	DRI (def; das)
DSRCT	JN-DSRCT-1	0.0625	0.101 ± 0.05	0.463	(9.30; 2.81)
		0.125	0.202 ± 0.08	0.517	(6.02; 2.85)
		0.25	0.411 ± 0.10	0.532	(4.16; 3.43)
		0.5	0.736 ± 0.08	0.485	(3.24; 5.69)
		1	0.849 ± 0.01	0.685	(2.02; 5.23)
		2	0.870 ± 0.01	1.264	(1.07; 3.04)
		4	0.873 ± 0.01	2.500	(0.54; 1.56)

ES	TC32	0.0625	0.366 ± 0.06	0.576	(12.7; 2.01)
		0.125	0.409 ± 0.13	0.783	(7.28; 1.55)
		0.25	0.484 ± 0.17	0.844	(4.56; 1.60)
		0.5	0.562 ± 0.15	0.939	(2.87; 1.69)
		1	0.671 ± 0.12	0.885	(2.02; 2.56)
		2	0.758 ± 0.07	1.000	(1.39; 3.56)
		4	0.774 ± 0.07	1.802	(0.74; 2.20)

ARMS	Rh30	0.0625	0.155 ± 0.16	0.334	(18.8; 3.57)
		0.125	0.176 ± 0.09	0.514	(9.93; 2.42)
		0.25	0.246 ± 0.14	0.515	(6.16; 2.84)
		0.5	0.440 ± 0.12	0.332	(4.73; 8.30)
		1	0.629 ± 0.03	0.342	(3.44; 19.5)
		2	0.741 ± 0.02	0.486	(2.22; 27.9)
		4	0.704 ± 0.02	1.088	(1.02; 9.62)

ERMS	RD	0.0625	0.239 ± 0.03	0.083	(22.9; 25.6)
		0.125	0.218 ± 0.03	0.177	(10.7; 11.9)
		0.25	0.281 ± 0.02	0.291	(6.50; 7.29)
		0.5	0.415 ± 0.03	0.411	(4.56; 5.23)
		1	0.557 ± 0.04	0.587	(3.15; 3.70)
		2	0.720 ± 0.03	0.876	(1.55; 4.37)
		4	0.758 ± 0.03	1.564	(0.86; 2.46)

DSRCT: desmoplastic small round cell tumor; ES : Ewing sarcoma; ARMS: alveolar rhabdomyosarcoma; ERMS: embryonal rhabdomyosarcoma; FA-value: the fraction of cell viability affected by treatment; CI: combination index; DRI: dose reduction index; def: defactinib; das: dasatinib.

## Data Availability

Data are available on request to the corresponding author.

## Supplementary Materials

Supplementary Materials Description Supplementary Material. 2.1 Immunohistochemistry (IHC) and statistical analysis. 2.5 Western blot. Figure S1: *pFAK and pSrc expressions*. An example of the intensity of pFAK and pSrc staining in tumor tissue assessed by immunohistochemistry (IHC). Intensity is subdivided into negative (0), weak-positive (1), positive (2), and high-positive (3) expressions. ARMS tumor tissue is used as an example. Images were taken at 40x magnification. Figure S2: *pFAK and pSrc expressions and effects of defactinib and dasatinib single-agent treatment in EW8 (ES), Rh41 (ARMS), and Rh18 (ERMS) cells.* (a) Baseline pFAK (Tyr397) and pSrc (Tyr416) expressions in the EW8, Rh41, and Rh18 cell lines. (b) Cell viability (%) following defactinib and dasatinib single-agent treatment in the EW8, Rh41, and Rh18 cell lines. Figure S3: *quantification of pFAK, pSrc, and γH2AX expressions.* Quantification of (a) FAK, (b) pFAK (Tyr397), (c), Src, (d) pSrc (Tyr416), and (e) *γ*H2AX (Ser139) expressions after 24 h single-agent and combination treatment. (p)FAK, (p)Src, and *γ*H2AX expressions are depicted as a percentage of the loading control GAPDH. Figure S4: *effects of defactinib and dasatinib combination treatment* (a–c). Cell viability (%) following defactinib and dasatinib simultaneous, constant-ratio combination treatment in the Rh18 (a), Rh41 (b), and EW8 (c) cell lines alongside the corresponding isobologram, representing the level of drug synergy. The X- and Y-axes of the isobologram represent the fraction of the portion of the drug in the combination treatment (D_1_ + D_2_) necessary to reduce an x% cell viability (D_1/2_) divided by the dose necessary as a single agent to generate a reduction of a similar x% cell viability (D_X_)_1/2_. D_1_ = defactinib, and D_2_ = dasatinib. The line connecting the X- and *Y*-axes represents an additive effect (CI = 1). Points left of the line are considered synergistic (CI < 1.0). Table S1. FA, CI, and DRI values for defactinib and dasatinib combination treatment in EW8, Rh41, and Rh18 cell lines.
