# Supplementary material for: Targeting the FAK-Src Complex in Desmoplastic Small Round Cell Tumors, Ewing Sarcoma, and Rhabdomyosarcoma
Source: Sarcoma. 2022 May 11;2022:3089424. doi: 10.1155/2022/3089424 (PMC9153931; doi:10.1155/2022/3089424)
Supplement: Supplementary Materials — Description Supplementary Material. 2.1 Immunohistochemistry (IHC) and statistical analysis. 2.5 Western blot. Figure S1: pFAK and pSrc expressions. An example of the intensity of pFAK and pSrc staining in tumor tissue assessed by immunohistochemistry (IHC). Intensity is subdivided into negative (0), weak-positive (1), positive (2), and high-positive (3) expressions. ARMS tumor tissue is used as an example. Images were taken at 40x magnification. Figure S2: pFAK and pSrc expressions and effects of defactinib and dasatinib single-agent treatment in EW8 (ES), Rh41 (ARMS), and Rh18 (ERMS) cells. (a) Baseline pFAK (Tyr397) and pSrc (Tyr416) expressions in the EW8, Rh41, and Rh18 cell lines. (b) Cell viability (%) following defactinib and dasatinib single-agent treatment in the EW8, Rh41, and Rh18 cell lines. Figure S3: quantification of pFAK, pSrc, and γH2AX expressions. Quantification of (a) FAK, (b) pFAK (Tyr397), (c), Src, (d) pSrc (Tyr416), and (e) γH2AX (Ser139) expressions after 24 h single-agent and combination treatment. (p)FAK, (p)Src, and γH2AX expressions are depicted as a percentage of the loading control GAPDH. Figure S4: effects of defactinib and dasatinib combination treatment (a–c). Cell viability (%) following defactinib and dasatinib simultaneous, constant-ratio combination treatment in the Rh18 (a), Rh41 (b), and EW8 (c) cell lines alongside the corresponding isobologram, representing the level of drug synergy. The X- and Y-axes of the isobologram represent the fraction of the portion of the drug in the combination treatment (D1 + D2) necessary to reduce an x% cell viability (D1/2) divided by the dose necessary as a single agent to generate a reduction of a similar x% cell viability (DX)1/2. D1 = defactinib, and D2 = dasatinib. The line connecting the X- and Y-axes represents an additive effect (CI = 1). Points left of the line are considered synergistic (CI < 1.0). Table S1. FA, CI, and DRI values for defactinib and dasatinib combination treatment in [file 3089424.f1.docx]

Title page

**Targeting the FAK-Src complex in Desmoplastic Small Round Cell Tumors, Ewing sarcoma and Rhabdomyosarcoma**

*Anke E.M. van Erp^1^, Melissa H.S. Hillebrandt-Roeffen^1^, Niek F.H.N. van Bree^1^, Tim A. Plüm^1^, Uta. E. Flucke^2^, Emmy D.G. Fleuren^3^, Winette T.A. van der Graaf ^4^, Yvonne M.H. Versleijen-Jonkers^1*^*

^1^Department of Medical Oncology, Radboud University Medical Center, P.O. Box 9101, 6500HB Nijmegen, The Netherlands, <mailto:yvonne.versleijen-jonkers@radboudumc.nl>

^2^Department of Pathology, Radboud University Medical Center, P.O. Box 9101, 6500HB, Nijmegen, The Netherlands

^3^Children's Cancer Institute Australia, Lowy Cancer Research Centre, University of New South Wales, Sydney, NSW, Australia

^4^Department of Medical Oncology, The Netherlands Cancer Institute – Van Leeuwenhoek, 1066CX Amsterdam, The Netherlands

*Running title:* Targeting the FAK-Src complex in sarcoma

*^*^Corresponding author:*

Yvonne M.H. Versleijen-Jonkers

Department of Medical Oncology (internal postal code: 452)

Radboud University Medical Center

P.O. Box 9101

6500 HB Nijmegen, the Netherlands

Phone: 0031-(0)24-3618897

E-mail: [yvonne.versleijen-jonkers@radboudumc.nl](mailto:yvonne.versleijen-jonkers@radboudumc.nl)

ORCID: 000-0001-7625-0505

**2. Supplementary materials and methods**

*2.1 Immunohistochemistry (IHC) and statistical analysis*

FAK, pFAK, Src and pSrc IHC was performed on 4µm thick, formalin-fixed, paraffin-embedded (FFPE) tissue sections or TMAs (core size 1 or 2 mm) and RD/JN-DSRCT-1 tumor xenografts. Caspase-3 and γH2AX IHC was performed on the tumor xenografts. Sections were deparaffinized in xylol and rehydrated through a graded ethanol into water series. Antigen retrieval was performed by heating the slides in EDTA buffer, pH9 for 10min or 15min at 100°C (pSrc/pFAK and Src respectively), or by heating the slides in citrate buffer, pH6 for 10 min at 100°C (FAK and γH2AX). Endogenous peroxidase activity was blocked with 3% H_2_O_2_ in distilled water for 10min at room temperature (RT). Subsequently, sections were incubated with rabbit monoclonal anti-FAK (1:200, #71433), anti-phosphorylated FAK (Tyr397) (1:100, #8556), anti-Src (1:2400, #2109) anti-phosphorylated Src (Tyr416) (1:50, #2101), and anti-phosphorylated Histone H2A.X (Ser139) (1:250, #9718)(Cell Signaling Technology, Leiden, the Netherlands) in antibody diluent in a humidified chamber overnight at 4°C. Next, tissue sections were incubated with Poly-HRP-GAMs/Rb IgG (ImmunoLogic, Duiven, the Netherlands) in EnVision™ FLEX Wash Buffer (Dako, Agilent, Amstelveen, the Netherlands) (1:1) for 30min at RT. Antibody binding was visualized using the EnVision™ FLEX Substrate Working Solution (Dako) for 10min at RT. Finally, slides were counterstained with haematoxylin, dehydrated and coverslipped. Caspase-3 staining was performed as described previously (Fleuren ED et al. IJC 2014;135(12):2770-82).

Slides were scored for FAK, pFAK, Src, pSrc, caspase-3 and yH2AX expression by two independent observers. (p)FAK and (p)Src expression levels were evaluated as follows: 0 = no positive cells, 1 = weak expression, 2 = positive expression and 3 = highly positive expression, and the percentage of tumor tissue positively stained was determined. An example of each pSrc/pFAK staining intensity is given in Fig S1. H-scores were calculated as follows: staining intensity (0-3) * % tumor tissue positively stained (0-100). H-scores equal to 0, ≤ 50, 51-100 and >100 are considered negative ((p)FAK/(p)Src^neg^), weak ((p)FAK/(p)Src^weak^), positive ((p)FAK/(p)Src^pos^) and high-positive expression ((p)FAK/(p)Src^high^), respectively.

For the γH2AX staining on JN-DSRCT-1 and RD mouse tumors, positive cells in 10 high power fields (HPF) at 160x magnification were counted and subdivided in three categories: < 10 (1), 10–50 (2) or ≥ 50 positive cells (3). For Caspase-3 staining 15 nonoverlapping fields per section were counted at 160x magnification. The percentage of positive cells as proportion of all counted cells was calculated and used for analysis as described before (Fleuren ED et al. IJC 2014;135(12):2770-82). Mean intenstity scores were calculated per group and a Student’s t-test was used to compare differences between treatment groups.

Digital images were generated with VisionTek^TM^ (Sakura, version 2.6) and analyzed at 40 or 80x magnification.

The prognostic value of pFAK, pSrc and concurrent pFAK and pSrc expression in DSRCT, ES and RMS tumor tissue was examined by assessment of the relation between categorical parameters using Chi-square or Fisher’s exact test and by using the Kaplan-Meier method (Log rank test) to assess associations with overall and/or event-free survival (OS and EFS). Statistical analysis was performed using IBM SPSS Statistics 22 and p-values <0.05 were considered statistically significant.

*2.5. Western Blot*

Monoclonal rabbit anti-FAK (1:1000, #71433), anti-phosphorylated FAK (Tyr397) (1:1000, #8556), anti-Src (1:1000, #2109), anti-phosphorylated Src (Tyr416) (1:500, #2101), and anti-phosphorylated Histone H2A.X (Ser139) (1:1000, #9718) were purchased from Cell Signaling Technology (Leiden, the Netherlands). Loading control monoclonal mouse anti-α-tubulin (1:1000, #A11126) or anti-GAPDH (1:10000, #Ab8245) were purchased from Thermo Scientific (Breda, the Netherlands) or Abcam (Cambridge, UK), respectively.

All Western blot experiments were performed in duplicate.


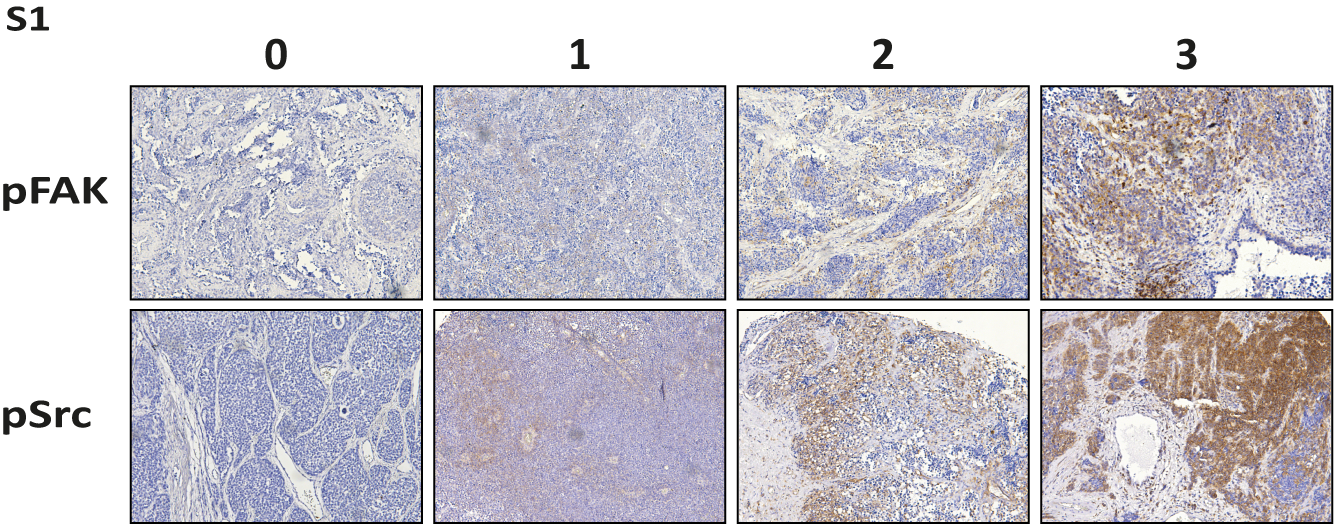


**Fig. S1** *pFAK and pSrc expression*

An example of the intensity of pFAK and pSrc staining in tumor tissue assessed by immunohistochemistry (IHC). Intensity is subdivided in negative (0), weak positive (1), positive (2) and high positive (3) expression. ARMS tumor tissue is used as an example. Images were taken at 40x magnification.


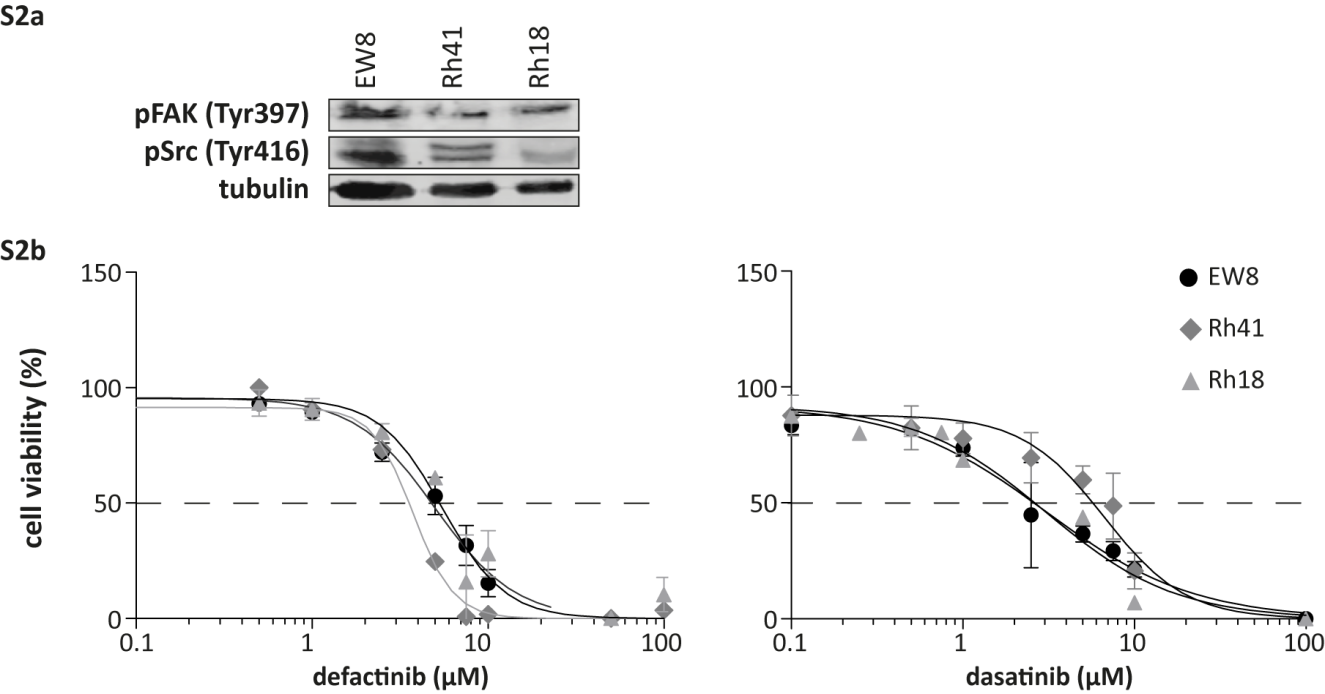


**Fig. S2** *pFAK, pSrc expression and effects of defactinib and dasatinib single-agent treatment in EW8 (ES), Rh41 (ARMS) and Rh18 (ERMS) cells*

(A) Baseline pFAK (Tyr397) and pSrc (Tyr416) expression in the EW8, Rh41 and Rh18 cell line. (B) Cell viability (%) following defactinib and dasatinib single-agent treatment in the EW8, Rh41 and Rh18 cell line.


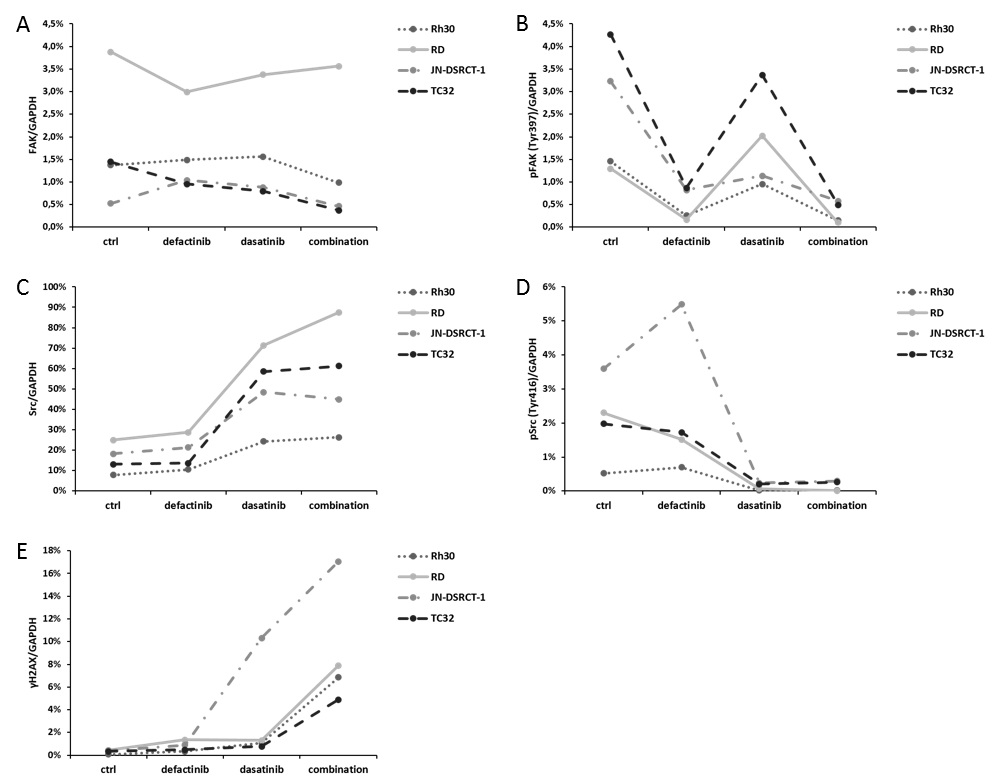


**Fig. S3** *Quantification of (p)FAK, (p)Src and γH2AX expression* Quantification of (A) FAK, (B) pFAK (Tyr397), (C), Src, (D) pSrc (Tyr416) and (E) γH2AX (Ser139) expression after 24h single agent and combination treatment. (p)FAK, (p)Src and γH2AX expression is depicted as a percentage of the loading control GAPDH.


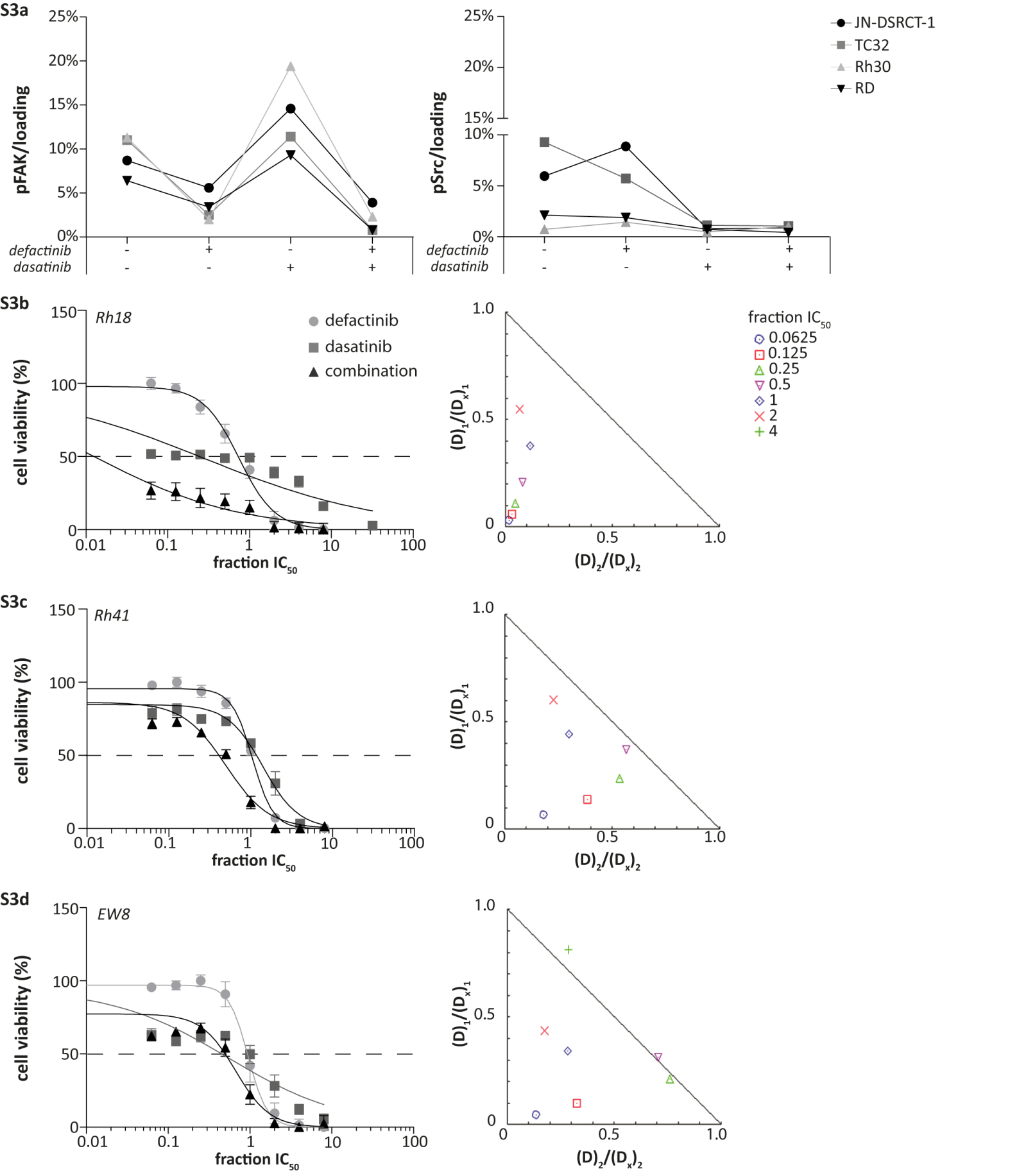


**A**

**B**

**C**

Table S1 **FA-, CI- and DRI-values for defactinib and dasatinib combination treatment in EW8, Rh41 and Rh18 cell line.**

**Fig. S4** *Effects of defactinib and dasatinib combination treatment*

(A-C) Cell viability (%) following defactinib and dasatinib simultaneous, constant-ratio combination treatment in the Rh18 (A), Rh41 (B) and EW8 (C) cell line alongside the corresponding isobologram, representing the level of drug synergy. The X- and Y-axis of the isobologram represent the fraction of the portion of the drug in the combination treatment (D_1_+D_2_) necessary to reduce an x% cell viability (D_1/2_) divided by the dose necessary as a single-agent to generate reduction of a similar x% cell viability (D_X_)_1/2_. D_1_ = defactinib, D_2_ = dasatinib. The line connecting the X- and Y-axis represents an additive effect (CI = 1). Points left of the line are considered synergistic (CI < 1.0).

Table S1 **FA-, CI- and DRI-values for defactinib and dasatinib combination treatment in EW8, Rh41 and Rh18 cell line.**

| *Subtype* | *Cell line* | *Fraction IC_50_* | *FA-value*  *(mean ± SD)* | *CI* | *DRI (def;das)* |
| --- | --- | --- | --- | --- | --- |
| ES | **EW8** | 0.0625 | 0.305 ± 0.02 | 0.182 | (21.6;7.38) |
|  |  | 0.125 | 0.280 ± 0.03 | 0.426 | (10.1;3.06) |
|  |  | 0.25 | 0.260 ± 0.05 | 0.973 | (4.74;1.31) |
|  |  | 0.5 | 0.368 ± 0.08 | 1.021 | (3.17;1.42) |
|  |  | 1 | 0.626 ± 0.09 | 0.625 | (2.92;3.54) |
|  |  | 2 | 0.784 ± 0.04 | 0.611 | (2.29;5.75) |
|  |  | 4 | 0.805 ± 0.03 | 1.098 | (1.23;3.50) |
| ARMS | **Rh41** | 0.0625 | 0.238 ± 0.05 | 0.248 | (14.9;5.52) |
|  |  | 0.125 | 0.228 ± 0.04 | 0.524 | (7.24;2.59) |
|  |  | 0.25 | 0.289 ± 0.04 | 0.774 | (4.18;1.87) |
|  |  | 0.5 | 0.414 ± 0.05 | 0.938 | (2.69;1.77) |
|  |  | 1 | 0.691 ± 0.06 | 0.743 | (2.26;3.33) |
|  |  | 2 | 0.839 ± 0.02 | 0.830 | (1.66;4.41) |
|  |  | 4 | 0.841 ± 0.02 | 1.645 | (0.83;2.24) |
| ERMS | **Rh18** | 0.0625 | 0.538 ± 0.07 | 0.047 | (33.1;59.1) |
|  |  | 0.125 | 0.543 ± 0.08 | 0.092 | (16.8;31.0) |
|  |  | 0.25 | 0.575 ± 0.08 | 0.156 | (9.18;21.2) |
|  |  | 0.5 | 0.593 ± 0.07 | 0.286 | (4.83;12.6) |
|  |  | 1 | 0.623 ± 0.06 | 0.496 | (2.64;8.53) |
|  |  | 2 | 0.724 ± 0.05 | 0.627 | (1.82;14.7) |
|  |  | 4 | 0.729 ± 0.05 | 1.225 | (0.93;6.82) |
| *ES: Ewing sarcoma; ARMS: alveolar rhabdomyosarcoma; ERMS: embryonal rhabdomyosarcoma; FA-value: the fraction of cell viability affected by treatment; CI: combination index; DRI: dose reduction index; def: defactinib; das: dasatinib* | | | | | |
